# Gleaning Structure from Sound: The Role of Prosodic Contrast in Learning Non-adjacent Dependencies

**DOI:** 10.1007/s10936-016-9412-8

**Published:** 2016-02-09

**Authors:** Ileana C. Grama, Annemarie Kerkhoff, Frank Wijnen

**Affiliations:** Department of Humanities, Utrecht Institute of Linguistics OTS, Utrecht University, Trans 10, 3512 JK Utrecht, The Netherlands

**Keywords:** Statistical learning, Non-adjacent dependencies, Prosody, Gestalt principles, Language acquisition

## Abstract

The ability to detect non-adjacent dependencies (i.e. between *a* and *b* in *aXb*) in spoken input may support the acquisition of morpho-syntactic dependencies (e.g. *The princess* ***is*** *kiss* ***ing*** *the frog*). Functional morphemes in morpho-syntactic dependencies are often marked by perceptual cues that render them distinct from lexical elements. We use an artificial grammar learning experiment with adults to investigate the role of perceptual cues in non-adjacent dependency learning, by manipulating the perceptual/prosodic properties of the *a* / *b* elements in *aXb* strings and testing participants’ incidental learning of these dependencies. Our results show that non-adjacent dependencies are learned both when the dependent elements are perceptually prominent, *and* when they are perceptually reduced compared to the intervening material (in the same way that functional words are reduced compared to lexical words), but only if integrated into a natural prosodic contour. This result supports the idea that the prosodic properties of natural languages facilitate non-adjacent dependency learning.

## Introduction

It is a core property of human languages that they exhibit dependencies: patterns of co-occurrence between linguistic units or classes of units (if *a* occurs, *b* usually occurs too) which indicate underlying rules/regularities (*a* selects for *b*). Thus, dependencies between linguistic units (phonological, morphological, syntactic, etc.), either adjacent (*ab*) or non-adjacent (*aXb*), can prove highly informative to a naive learner. Dependencies between adjacent syllables (e.g. the high co-occurrence probability of syllables *ba* and *by*, forming the word *baby*, versus the low co-occurrence probability of syllables *ty* and *ba* indicating a word boundary in the phrase *pretty baby*) can facilitate the segmentation of words from continuous speech streams (Saffran et al. 1996). Dependencies between adjacent (classes of) morphemes indicate subcategorization properties, such as the article *the* selecting only nouns or noun phrases as its complements. Several studies have demonstrated learners’ ability to pick up co-occurrence patterns between adjacent units (Saffran et al. 1996; Aslin et al. 1998) or classes of units (Gómez and Lakusta 2004; Gerken et al. 2005; Reeder et al. 2013) from artificial grammars that they are exposed to in controlled lab settings.

Another type of dependencies that can be observed in natural languages are co-occurrence patterns between non-adjacent morphemes, which often indicate syntactic relationships like agreement (1,3) or verbal aspectual paradigms (2):

**Table:** 

(1) a. Noi toţi greşim câteodată.	(Romanian)
We all err.$\underline{1^{\mathrm{st}}\hbox {pl}}$ sometimes	
b. Voi toţi greşiţi câteodatã	
You all err.$\underline{2^{\mathrm{nd}}\hbox {pl}}$ sometimes	
(2) Ik heb vandaag de dokter gebeld.	(Dutch)
I have today the doctor PART.call	
(3) una bella ragazza / un bel ragazzo	(Italian)
a.fem beautiful.fem girl.fem / a.masc beautiful.masc boy.masc	

Suppose a naive learner were exposed to examples such as (1): if this learner possessed the ability to detect the high co-occurrence rate between the morphemes *noi* and *-im*, s/he could infer that the presence of one morpheme predicts (the form of) the other. With exposure to the full paradigm of dependencies between subject pronouns and verb endings (*noi_-im*, *voi_-iţi*, etc.), the learner might infer that the morphological forms of the items in those particular positions (i.e. subject and verb-suffix) are always correlated, and that, therefore, there must be a syntactic relationship between the two items.^1^ Thus, by observing surface properties of the input such as co-occurrence patterns between specific items, one could arguably infer more abstract morpho-syntactic rules of natural languages.

Around 18 months, children have been shown to be sensitive to morpho-syntactic dependencies in their native language: when exposed to alternating passages with correct or incorrect dependencies (e.g. *The cook* *is* *always bak* *ing* *bread* vs. *The cook *can* *always bak* *ing* *bread*), they show a reliable preference for the correct ones (van Heugten and Johnson 2010; van Heugten and Shi 2010; Höhle et al. 2006; Santelmann and Jusczyk 1998; Wilsenach and Wijnen 2004). Thus, at the age of 18 infants seem to become aware of the morphemes that engage in dependencies *and* of the one-to-one correspondence between them. However, languages vary both with respect to marking these morpho-syntactic relationships overtly or not (e.g. Italian marks gender agreement within the noun phrase, as in (3), whereas English does not) and what specific morphemes are used to mark these relationships. Therefore, infants must learn from surface properties of the input about the specific morphemes that enter syntactic relationships, as well as the specific one-to-one pairings between them.

A working theory in the field of psycholinguistics is that learners identify non-adjacent dependencies (NADs) by tracking co-occurrence statistics between the non-adjacent elements, in the same way that they track co-occurrence statistics between adjacent elements (Pacton and Perruchet 2008). Thus, learners will detect a dependency between *a* and *b* if there is a high probability that when item *a* occurs in the input item *b* will follow, either adjacently (*ab*) or non-adjacently (*aXb*). Artificial Grammar Learning (AGL) studies have investigated this learning mechanism by examining adults’ or infants’ ability to detect NADs in strings of unfamiliar speech, which exhibit a systematic co-occurrence between a token $a_{\mathrm{i}}$ and a token $b_{\mathrm{i}}$, separated by a variable token $X (a_{i}Xb_{i}$, where the form of $a_{\mathrm{i}}$ predicts the form of $b_{\mathrm{i}}$ with 100 % probability). These studies find that, after familiarization with an *aXb* language (consisting of nonsense strings such as *pel kicey jic*), adult learners show a reliable preference for consistent $a_{i}\_b_{i}$ dependencies over inconsistent $a_{i}\_b_{j}$ ones (where the final element was not predicted by the first; e.g. Endress and Bonatti 2006; Endress and Mehler 2009; Gómez 2002; Newport and Aslin 2004; Onnis et al. 2004; Peña et al. 2002). Infants at 12–18 months are also able to discriminate consistent vs. inconsistent dependencies, by displaying significantly different looking times to the two types of stimuli in a Headturn Preference Procedure (Gómez 2002; Gómez and Maye 2005; Lany and Gómez 2008; Kerkhoff et al. 2013).

Apparently, learners should be able to track co-occurrence patterns between any two items in a string. However, Newport and Aslin (2004) point out that a mechanism tracking transitional probabilities between any two units in a string, adjacent or non-adjacent, would meet with a combinatorial explosion, as the number of possible pairs grows exponentially with the length of the string. For every string with n elements, there would be n*(n $+$ 1)/2 potential dependencies (n $-$ 1 of which would be adjacent, and the rest (n $-$ 2)*(n $+$ 1)/2 non-adjacent). But a learning mechanism faced with a combinatorial explosion is bound to be ineffective, as it memorizes and processes countless pairs which may turn out not to be dependencies in the end. A more efficient mechanism would limit the amount of computations by only selecting a subset of the total amount of possible dependencies.

Indeed, it has been shown that there may be various constraints on the non-adjacent dependency-tracking mechanism, limiting the number of units over which non-adjacent statistics are computed. Several studies have shown that dependencies are learned between elements (*a_b*) with markedly different properties from the intervening material (*X*). For instance, Gómez (2002) showed that participants detected NADs in (isolated) $a_{i} Xb_{i}$ strings only when the intervening word *X* varied sufficiently (i.e. the three $a_{i}\_b_{i}$ dependencies were learned better with 24 different *X*s than with 12 or 6). Learners acquired the dependencies better when the distributional frequency and stability of the dependent elements contrasted with the variability of the intervening elements.

Dependent elements can also have different phonological properties than the intervening material. van den Bos et al. (2012) and Onnis et al. (2005) showed that NAD-learning was facilitated by phonological cues marking the non-adjacent pairs as distinctive—for instance if dependent syllables began with a plosive consonant while intervening syllables began with a continuant. Newport and Aslin (2004) showed that, in a continuous string of syllables, participants could learn dependencies of vowels over consonants, or vice-versa, but not of syllables over syllables, suggesting that dependencies where only learnable when the dependent elements were segmentally distinctive from their environment.

In all these studies dependent elements had to ‘stand out’, either due to their invariability (frequency) or their perceptually distinct nature. Newport and Aslin have proposed that the detection of NADs relies on Gestalt principles of similarity: non-adjacent elements that are similar to each other but distinct from the intervening material are grouped and processed together. For instance, in a stream of speech with a pitch contour consisting of peaks and troughs, the high-pitched elements will be grouped together, and represented separately from the low-pitched elements, and the co-occurrence statistics between (non-adjacent) high-pitched elements will be easier to compute than between a high-pitched and a low-pitched (adjacent) element. This principle has been shown to apply domain generally, to linguistic as well as non-linguistic (musical tones, noises) domains (see Creel et al. 2004 and Gebhart et al. 2009). According to the Gestalt hypothesis, therefore, the NAD-learning mechanism is most efficient when there is some cue that groups dependent elements as similar to each other and distinct from the intervening/surrounding material. Newport and Aslin make no claims about the specific level at which these Gestalt principles could apply: their own studies reveal Gestalt perception effects at the linguistic level of segmental phonology (Newport and Aslin 2004) as well as the acoustic level of pitch-perception (Creel et al. 2004).

### NAD-Learning and Natural Language

If NAD-learning is a highly constrained mechanism, it could only be a valid mechanism for learning natural languages if its constraints are satisfied in natural languages. For instance, if the variability of the intervening material is a crucial constraint on NAD-learning (Gómez 2002) then dependencies in natural languages should also be instantiated between stable, frequent elements over highly variable ones. Indeed, natural languages seem to satisfy this constraint: morpho-syntactic dependencies [see (1)] are often instantiated between functional morphemes (pronouns, auxiliaries, inflectional morphology), spanning lexical elements (nouns, verbs, etc.). While functors in every language are frequent and relatively invariable, lexical elements are drawn from a large, open class, and are therefore highly variable and less frequent. Distributional properties may thus make functional elements ‘stand out’ in a way that facilitates the detection of co-occurrence patterns between them.

Functional morphemes are distinguishable from their lexical counterparts by more than just distributional properties. Shi et al. (1998) analyzed child-directed speech in Turkish and Mandarin Chinese, and showed that functors have distinctive distributional, acoustic and phonological properties: not only are they more frequent, they are shorter in duration with lower relative amplitude, and have simpler syllabic structure. This constellation of cues leads to 80–90 % accuracy in categorizing an element as functional or lexical. Monaghan et al. (2007) took four different languages (English, Dutch, French and Japanese), and showed that an even larger constellation of cues (length, syllabic complexity, manner and place of articulation of consonants, vowel density, vowel reduction, vowel position) distinguished between functional and lexical items. Furthermore, it has been shown that newborns can discriminate between functional and lexical words based on perceptual cues alone (Shi et al. 1999). Functors are thus marked by perceptual cues that make them distinctive, and this distinctiveness is picked up reliably by learners from the very beginning. But if functors themselves are easy to identify, does this render dependencies between them also easier to detect/learn?

### Purpose of the Present Study

In this study we investigate *whether* perceptual cues affect the detection of NADs, and if so, *how*. The Gestalt principles of perception hypothesis, put forth by Newport and Aslin (2004), predicts that the specific perceptual cues that mark functional words/morphemes in natural languages will facilitate the detection of co-occurrence patterns between ‘functional’-sounding elements (over ‘lexical’-sounding ones) in a controlled learning environment such as an AGL paradigm. We employ just such a paradigm to investigate the role of perceptual *distinctiveness* in the detection of non-adjacent dependencies. According to the Gestalt principles of perception hypothesis (henceforth Hypothesis 1), dependent elements that are perceptually distinct but similar to each other are represented and computed together on a separate level, and therefore patterns between them are more easily detected. Hypothesis 1 predicts that dependencies between perceptually ‘reduced’ (functional) morphemes, spanning ‘lexical’-sounding material, should be easily acquired due to the perceptual distinctiveness of functors. According to this hypothesis, then, learning dependencies in natural languages should also be enabled by the specific perceptual distinction between the functional/lexical class, which allows functors to be represented on a separate level and facilitates the discovery of patterns between them.

Note, however, that functors are distinctive by being less acoustically prominent than the elements around them. Data from L1 acquisition suggests that this also makes them harder to track in spoken input: infants prefer listening to lexical over functional items in their native language (Shi and Werker 2001, 2003), and have difficulties with the phonological encoding of function words (Hallé et al. 2008; Shi et al. 2006a, b; Shi and Lepage 2008), especially the less acoustically salient ones (Strömqvist et al. 2001). It is possible that this lack of acoustic salience will make learners (adults or infants) less likely to focus on the target elements, or detect the dependencies between them. An alternative to Hypothesis 1, therefore, is that NAD-learning is reliant on the (acoustic) *prominence* (rather than the distinctiveness) of dependent elements: the more prominent (i.e. higher in pitch, intensity, longer in duration) the elements, the easier it is to keep track of them (Strömqvist et al. 2001), and therefore the easier it is to detect patterns between them. This predicts that dependencies between functional-sounding (over lexical-sounding) elements would be difficult to detect because functors themselves are not perceptually prominent.

If Gestalt principles of perception are used to group functor-like units together and compute dependencies between them over lexical-like units, the next question is *how* exactly these grouping principles are used, and what is the *nature* of the cues they rely on to distinguish between different types of units. Shi et al. (1998) as well as Monaghan et al. (2007) combine a variety of cues from the linguistic (segmental phonology, syllable structure, etc.) as well as non-linguistic (acoustic) domain to mark the lexical/functional distinction. Functors may be distinct from lexical words at a purely *acoustic* level (e.g. pitch, amplitude, etc.), or these acoustic differences can be exploited at a higher, linguistic level of analysis: *prosody*. Functional words and morphemes are often prosodic clitics (Selkirk 1996), and are therefore prosodically unmarked compared to their lexical counterparts, which can receive lexical stress or tonal accent.

Do Gestalt principles of perception operate at the primary level of acoustic perception, grouping together or dissociating elements based on their acoustic properties, as has been shown in studies of NAD-learning with non-linguistic input (Creel et al. 2004; Gebhart et al. 2009)? Or do they also operate at a more domain-specific level, exploiting more abstract levels of linguistic representation, such as segmental phonology (Newport and Aslin 2004; Onnis et al. 2005) or prosody? Is NAD-detection with linguistic input constrained by acoustic or linguistic (prosodic) factors? The findings of Newport and Aslin (2004), Onnis et al. (2005) and van den Bos et al. (2012) reported above suggest that Gestalt principles guiding NAD-learning can apply at the level of phonological analysis. Arguably, if NAD-learning is to be a powerful tool for language acquisition, it should rely on linguistic representations, which are more abstract, robust in the face of variation or noise conditions, and more easily encoded in memory. Purely acoustic cues may guide NAD-learning of non-linguistic stimuli (Creel et al. 2004; Gebhart et al. 2009) but the acoustic factors identified by Shi et al. (1998) and Monaghan et al. (2007) to distinguish between functional and lexical elements could at least in part correlate with the different prosodic properties that these two categories have. Therefore, Hypothesis 2 of our study states that if Gestalt principles of perception facilitate the detection of dependencies between functor-like over lexical-like elements, then these principles must operate at the level of prosodic representation of the input: prosodically reduced elements are grouped together and dissociated from prosodically marked elements.

Previous studies have looked into the role of prosody in NAD-learning. Peña et al. (2002) and Endress and Bonatti (2006) (as well as Marchetto & Bonatti, 2013, for 18 month-olds) showed that inserting subtle segmentation cues (25 ms pauses) at the boundaries of *aXb* strings (as opposed to presenting them in a continuous stream) facilitated preference for ‘rule-words’ (novel *aX’b* strings). Langus et al. (2012) showed that phrase-final lengthening and intonational cues also facilitated a preference for rule-words, while Mueller et al. (2010) showed that segmentation pauses as well as a rising-falling pitch contour over the relevant chunks facilitated the detection of center-embedded dependencies $(a_{i} a_{j} b_{j} b_{i})$. Hence, prosody can cue segmentation of the input, facilitating the detection of dependency relations. However, is there a role to prosody that goes beyond segmenting and organizing the input? Can the prosodic status of individual words also facilitate the detection of dependencies between them?

### Research Questions and Implementation

In this study we aimed to test two Hypotheses:

#### **Hypothesis 1**

The detection of dependencies between functor-like words over lexically-sounding words is facilitated by Gestalt principles of perception which group the perceptually similar functor-like units together

#### **Hypothesis 2**

If Hypothesis 1 is correct, then Gestalt principles of perception operate at the level of prosodic representation of the input, grouping together prosodic ‘clitics’

To test Hypothesis 1 we examined the role of perceptual prominence vs. perceptual distinctiveness in a controlled AGL paradigm, by employing a simple artificial grammar (*aXb*, cf. Gómez 2002) where we varied the acoustic cues to the a_b dependencies across three different conditions (while maintaining the *X*s constant) and observed the effect on learning. Specifically, we tested three Acoustic Conditions: an Emphasized Condition, where the $a_{i}$ and $b_{i}$ elements in the $a_{i}\_b_{\mathrm{i}}$ dependencies were more acoustically prominent than the intervening *X*s, a Lexical Condition, where the dependent elements, like the *X*s, had the perceptual properties of lexical items in natural languages (e.g. lexical stress, full vowels, etc.), and a Functional Condition, where the dependents had perceptual properties of functors (reduced vowel, lower pitch, intensity and shorter duration than lexical words), and are therefore less prominent than the *X*s. If Hypothesis 1 is correct, participants should learn the dependencies well in the Emphasized and Functional Conditions, but perform poorest in the Lexical Condition, where the dependent elements are not perceptually distinctive. Conversely, if the dependencies’ salience is a function of their acoustic prominence performance will decline linearly over the three Conditions, and performance in the Functional Condition (where dependencies are least perceptually prominent) should be poorest. In both cases performance in the Emphasized Condition should be superior to performance in the Lexical Condition. If perceptual cues do not affect NAD-learning, performance should not vary across Conditions.

To address Hypothesis 2 and disentangle whether learners pick up dependencies based on the *acoustic* or *prosodic* similarity between the dependent elements, we constructed stimuli in such a way that dependent elements in the Emphasized and Functional Conditions were always marked by clear acoustic cues (pitch, duration and amplitude), that differentiated them from the intervening elements. The *prosodic* status of the words was determined by a combination of the acoustic cues (e.g. higher pitch was indicative of prosodic markedness), and (crucially!) the inter-word pause cues. For instance, Gómez (2002) employed 250 ms pauses between words in an $a_{i}Xb_{i}$ string, with strings being read out in a lively, child-friendly voice; these are audible pauses that normally mark boundaries between prosodic units in natural speech and would therefore be appropriate for delimiting (acoustically/prosodically) marked items, such as the *a* / *b* tokens in our Emphasized Condition. However, functional-sounding elements often have the prosodic status of clitics (Selkirk 1996), which means that they cannot be prosodic units of themselves but need to attach to a stem. We posited that shorter pauses, of 100 ms (which are below the auditory threshold for pause perception, cf. Zellner 1994, but are long enough to eliminate the need for co-articulation at the word boundaries^2^), would render the Functional Condition more natural.

We tested subjects in a $3\times 2$ between-subjects design, where each of the three Acoustic Conditions was tested with 2 Pause Versions: one with 100 ms pauses between the words in an *aXb* string (as if the string was a single prosodic unit), and one with 250 ms pauses between words (as if the *aXb* string was composed of three self-standing prosodic units). Thus, while in the Emphasized Condition with 250 ms or the Functional Condition with 100 ms pauses pause cues were consistent with acoustic cues in marking the prosodic status of the dependent *a* / *b* elements (as either accented or, respectively, reduced), the Emphasized Condition with the 100 ms pauses, as well as the Functional Condition with 250 ms pauses contained conflicting cues as to the prosodic status of *a* / *b*. For instance, in the Functional Condition with 250 ms pauses, *a* / *b* elements were recorded as prosodic clitics, but they were separated by the stem they should attach to by audible pauses.

If learners only relied on acoustic cues to mark the dependent elements *a* / *b* as similar to each other and distinct from the context, pause cues should be irrelevant to their learning performance and there should only be an effect of Acoustic Condition. If, on the other hand, learners employ Gestalt principles of perception on the *prosodic* level, then learning performance should decline in the conditions where acoustic vs. pause cues are inconsistent in marking the prosodic status of the *a* / *b* elements. In that case, we expect a significant Acoustic Condition by Pause Version interaction, better performance with 250 ms rather than 100 ms pauses for the Emphasized condition, and the reverse pattern for the Functional Condition.

## Experiment 1

We adopted the design of Gómez (2002) with stimuli (adapted to Dutch participants) from Grama et al. (2013). We tested NAD-learning in 3 different Conditions, as described above: Emphasized, Lexical and Functional, each with two Pause versions, 100 or 250 ms.Therefore, our design was a 3 (Acoustic Conditions) $\times$ 2 (Pause versions) full-factorial, between-subjects design. The methodology was the same across conditions: as in Gómez (2002), participants were exposed to a language consisting of 3 *a_b* dependencies combined exhaustively with a set of *X* elements. Following this familiarization, they were tested on their knowledge of the dependencies by receiving *aXb* strings either with correct (trained) or incorrect (untrained) dependencies and having to indicate, for each, whether they thought it was consistent with the language they had heard.

We introduced two important modifications to the methodology in Gómez (2002). Firstly, Gómez (2002) employed *aXb* test strings where the intervening *X* element was taken from the familiarization phase: thus, the correct test items, where the dependency was consistent with familiarization, were in fact *aXb* strings that had been heard in the familiarization. Previous literature has shown a distinction between the ability to recall chunks previously heard, and the ability to learn a given pattern as a *rule*, that is, to be able to use it productively and generalize it to novel contexts (Peña et al. 2002; Endress and Bonatti 2006; Endress and Mehler 2009, among others). In this study we are concerned with learner’s ability to obtain knowledge of non-adjacent dependencies as generalizable rules, and to be sensitive to these rules even when they are instantiated in unfamiliar contexts; this is the type of ability which will serve language acquisition in aiding learners to detect grammatical patterns as generalizable/productive rules of grammar, and not as patterns that occur in familiar contexts. In order to ensure that participants are not recalling chunks from familiarization but generalizing dependencies to novel contexts we have employed *aX’b* test strings where the intervening $X'$ element has not occurred during familiarization, ensuring that all test items were novel.

Secondly, in Gómez (2002) participants were familiarized with the artificial language with the explicit instruction that they should listen carefully because they would be subsequently tested on their knowledge of this language. This means participants were faced with a single task of listening intently, and could explicitly look for regularities that might improve their chances of success at test. However, in this study we aim to investigate a learning mechanism that may possibly aid first language acquisition. It is a general consensus in the literature that infants acquire their native language under incidental learning conditions: they do not benefit from explicit instruction, presumably direct their attention to the meaning rather than the structure of the input, and acquire language in an environment where various other stimuli or tasks may distract their attention (see Saffran et al. 1997, and references therein). Incidental learning in adults has been shown to closely resemble incidental learning in children, in a setup where participants were deterred from explicitly focusing on the spoken input by performing a simultaneous task of coloring (Saffran et al. 1997). Introducing a simultaneous task during an artificial grammar learning experiment has been shown to generally affect explicit, but not incidental learning (see Berry and Dienes 1997 and references therein), especially where the secondary task is in a different modality, or engages different computational mechanisms than the first. In short, adult incidental learning, induced by introducing a secondary, unrelated task, is likely to be a good model for a learning mechanism which might subserve early language acquisition. In this study we employ a coloring task, similar to Saffran et al. (1997).

### Participants

A group of 149 adult monolingual Dutch participants (17 males, age range 18–47, mean age 22) was recruited from the Utrecht Institute of Linguistics OTS database for adult participants and paid 5 euros for participation; a majority of them were students at the University, in other areas than linguistics. Participants were required to have no hearing impairment and no diagnosis of dyslexia or attention deficits. One participant (female) was excluded (from the Functional Condition with 250 ms pauses) for having previously participated in a similar experiment.

### Materials

*Familiarization*. In all three Conditions, two *aXb* (e.g. *tep naspu lut*) languages L1 and L2 were created, each with three $a_{i}\_b_{i}$ pairs (100 % conditional probability); language L1 contained the pairs *tep_lut*, *sot_jik *and *rak_toef*, whereas language L2 contained the pairs *tep_jik,* *sot_toef* and *rak_lut*, such that every $a_{i}\_b_{i}$ pair in one language was ungrammatical $({}^{*}\hbox {a}_{\mathrm{i}} \_\hbox {b}_{\mathrm{j}})$ in the other. A set of 18 different bisyllabic *X* words was used (see Table 2). During the familiarization, participants heard 324 strings (3a_b pairs $\times$ 18 Xs $\times$ 6 repetitions, randomized per participant), with 750 ms pauses between each two strings. We chose set size 18 for the intervening X elements because it has been shown to work just as well as set size 24 with infants (Gómez and Maye 2005), and we wanted to test if the same could be said for adults; if this set size is large enough to yield learning in some conditions but not others, it will highlight the effect of the perceptual cue manipulations that we wished to investigate. We chose bisyllabic intervening elements in line with Gómez (2002) to further facilitate the distinction between *a* / *b* tokens and *Xs*. As stated before, NADs have been shown to only be learned in cued contexts (Peña et al. 2002; Onnis et al. 2005), and in this experiment we cued dependent *a* / *b* elements both in terms of their high frequency/low variability and their (syllabic) length; if we do not obtain evidence of learning in some conditions, this must be due to the specific perceptual manipulations we introduced.

*Test*. Two novel *X*s were combined with the three $a_{i}\_b_{i}$ pairs of L1 (ungrammatical for learners of L2), and the three $a_{i} \_b_{i}$ pairs of L2 (ungrammatical for learners of L1), for a total of 12 test strings (6 of which were consistent with L1, and 6 were consistent with L2).

Stimuli for the Lexical and Functional Conditions were recorded in a sound-attenuated booth, at a sample frequency of 48 kHz, using a TASCAM DA-40 DAT-recorder. A female native Dutch speaker read out sentences in Dutch, each containing a nonce word, as naturally as possible. All *X*s, as well as Lexical *a* / *b*s, were recorded in the syntactic slot where a direct object noun would normally be found, in the template sentence:

**Table:** 

(4) Ik zie de ____ in de tuin.
I see the __ in the garden.
e.g. Ik zie de **fapoeg** in de tuin.
Ik zie de **tep** in de tuin

The *a* and *b* elements in the Functional Condition were recorded in the same carrier sentences, except they now filled the position of the determiner preceding the direct object (5); in all instances the speaker was instructed to realize the nonce words in accordance with their syntactic position:

**Table:** 

(5) Ik zie __ aapje in de tuin.
I see __ monkey in the garden
e.g. Ik zie **tep** aapje in de tuin.

For the Emphasized Condition, *a* and *b* elements were taken from Grama et al. (2013): the same female reader read out strings of four nonsense words (two intonational phrases, e.g. *[lotup tep] [poemer lut]*), in a lively manner, with emphasis on the monosyllabic words. Note that the methodology for recording stimuli for the Emphasized Condition is highly similar to that employed by Gómez (2002) for obtaining her stimuli (reading out the nonsense strings with lively, child-friendly intonation rendered the a/b elements in the string highly acoustically salient): the Emphasized Condition with 250 ms within-string pauses was therefore designed to approach Gómez (2002) as closely as possible, (with the exception of the methodological changes introduced, namely the secondary task designed to elicit implicit learning and the use of novel test items to test rule-learning).

**Table 1:** The a/b tokens, with IPA transcriptions and acoustic measures in Experiment 1

		Emphasized			Lexical			Functional		
		Mean pitch (Hz)	Mean amplitude (dB)	Duration (s)	Mean pitch (Hz)	Mean amplit. (dB)	Duration (s)	Mean pitch (Hz)	Mean amplit. (dB)	Duration (s)
a	TEP []	314.5	81.43	0.28	233.6	78.69	0.3	199	74.22	0.16
	SOT []	289.6	77.59	0.5	232.7	79.75	0.43	198.1	75.71	0.21
	RAK []	276.3	77.55	0.41	234.7	77.06	0.38	198.8	76.48	0.18
b	LUT []	289.1	81.82	0.47	234.1	79.2	0.38	200.2	78.27	0.17
	JIK [jIk]	293.8	81.27	0.43	232.7	79.46	0.37	197.8	78.89	0.18
	TOEF [tuf]	279.7	80.53	0.48	239.6	77.45	0.38	198.1	77.27	0.16
	Mean	290.5	80.03	0.428	234.5	78.6	0.373	198.7	76.81	0.177

To avoid large variations between acoustic properties of a/b tokens in a Condition, we resynthesized some of these tokens to match them for pitch and duration (the standard values were taken as the mean values of the original recordings): one element was shortened (i.e. functional jik, from 0.21 to 0.18 s) and 10 out of 12 were modified in pitch (four of the lexical variants and all of the functional variants). Elements in the Emphatic and Lexical Conditions were also scaled to an absolute peak of 0.99, whereas Functional a/bs were scaled to 0.85, resulting in lower amplitude values for Functional a/bs

**Table 2:** The intervening X elements, with IPA transcriptions and acoustic properties, used in Experiments 1–3

No.	*X*		Mean pitch (Hz)	Mean pitch of the first (stressed) syllable (Hz)	Mean intensity (dB)	Duration (s)	Duration of the first syllable (s)
	Familiarization	IPA					
1	blieker	[]	192.7	247.1	79.27	0.54	0.23
2	dufo	[dyfo]	166.1	250.2	79.99	0.57	0.2
3	fidang	[]	199.5	260.6	78.73	0.62	0.11
4	gopem	[]	173.4	247.7	79.29	0.65	0.18
5	kengel	[]	198.1	252.3	80.06	0.41	0.25
6	kijbog	[]	196.4	203.6	80.6	0.55	0.2
7	loga	[loxa]	182.1	244.5	79.89	0.55	0.27
8	malon	[]	195.9	255.9	81.78	0.61	0.24
9	movig	[movIx]	198.3	233.5	80.17	0.65	0.28
10	naspu	[]	180.3	272.7	79.41	0.55	0.27
11	nijfoe	[nuba]	197.8	261.7	81.41	0.6	0.28
12	noeba	[]	197.7	260	80.1	0.53	0.25
13	plizet	[]	191	253.9	80.71	0.59	0.26
14	rajee	[raje]	201.3	255	78.99	0.59	0.19
15	rogges	[]	203.9	265.8	78.44	0.6	0.21
16	seeta	[seta]	179.4	254.4	77.56	0.6	0.27
17	snigger	[]	175.1	265.3	76.56	0.58	0.26
18	wabo	[]	198.8	240	81.53	0.59	0.21
	Mean		190.43	251.34	79.69	57.67	0.23
	*Test*						
19	nilbo	[nIlbo]	204.9	236.9	81.46	0.65	0.19
20	pergon	[]	172.7	275	77.79	0.62	0.34

Acoustic measures, performed in Praat 5.3.03 (32-bit Edition for Windows, Boersma and Weenink 2005), of the a/b tokens for the three Acoustic Conditions are presented in Table 1, and show clear differences in acoustic properties between the three Conditions. Acoustic measures for the *X* elements employed for familiarization and test are presented in Table 2.

With these stimuli we ran a validation experiment in which we asked 9 naive listeners to match (on the basis of acoustic/prosodic similarity) *aX/bX* pairs, containing either lexical or functional *a* and *b* tokens, with real Dutch noun phrases, composed of either an adjective (lexical) and a noun or a determiner (functional) and a noun. For instance, in a two-alternative forced-choice task the participants would be given an *aX* pair in an ‘alien’ language (e.g. tep poemer) where the *a* sounded either functional or lexical, and would be asked to ’translate’ this alien phrase into either the phrase *een tijdschrift* (a newspaper, determiner + noun) or the phrase *oud tijdschrift* (old newspaper, adjective + noun), respectively. Participants correctly assigned experimental aX/bX pairs containing ‘functional’ or ‘lexical’*a* / *b* tokens to the targeted categories (determiner + noun or adjective + noun), in 79.6 % of the cases (SD $=$ 22.49). A one-sample *t* test confirmed that the accuracy score of 79.6 % was significantly above chance, $p = .004$. Thus, our artificial stimuli resembled the perceptual properties of Dutch functional and lexical elements.

### Procedure

*Familiarization.* Participants were seated in a sound-attenuated booth, coloring a mandala while listening to an ‘alien language’. They were instructed to ‘listen passively’ and attend primarily to the coloring. To avoid any motivation to explicitly look for patterns in the stimuli, participants were not informed of the subsequent test phase. The familiarization phase lasted between 10 and 15 min, depending on the Acoustic Condition and Pause version, and consisted of 324 *aXb* strings played out in a randomized order with 750 ms silences in between.

*Test phase*. After the familiarization, participants were told that the language they had heard had certain regularities related to word order and that they would hear 12 new sentences in this language, only six of which conformed to its rules. They would have to give grammaticality judgments for each of the strings based on their intuition. The test strings were presented in random order, and while each string played, a question appeared on the computer screen in front of them, asking: *Does this sentence belong to the language you have just listened to?* Note that the test strings had the same perceptual properties as the familiarization strings in each experiment, as the *a* / *b* tokens were identical to familiarization, and (novel) $X'$ tokens were recorded in the same way/session as the familiarization *Xs*. After hearing the test string participants responded ‘yes’ or ‘no’ by pressing one of two buttons on a button-box.

After the test, participants were debriefed on what they had noticed about the language they had heard, and what strategies they had used in answering the questions, if any. They were also asked to rate, on a scale from 1 to 7, their confidence in the responses they had given at test. According to the zero-correlation criterion (see Dienes 2007, and references cited therein) participants are implicit learners if their assessment of their own performance does not correlate with their actual performance. We wanted to see how implicit or explicit participants’ knowledge of the structure of the strings was: if participants who performed better on the test also expressed higher confidence in their answers, then there was some explicit awareness of the existence of structure in the input.

### Results

To assess learning performance in each of the six Conditions (3 Acoustic $\times$ 2 Pause), we ran One-Sample *t* tests on the mean Accuracy scores (percentage correct responses per participant) for each of the 6 cells, comparing each to chance (see Table 3). Participants in the Emphasized Condition with 250 ms pauses performed significantly above chance ($t (24) = 3.674, p = .001$), whereas learning did not reliably differ from chance expectation in any of the other 5 Conditions.

**Table 3:** Results for Experiments 1 and 2 per Acoustic Condition and Pause version, with number of participants, mean accuracy rates, *p* values for One-Sample *t* tests comparing mean accuracy rates to chance (and nonparametric, One-Sample Wilcoxon Signed-Rank Test for the New Functional Condition with 100 ms pauses), and effect size as Cohen’s d

	Emphasized	Lexical	Functional	New functional
250 ms				
No.	N $=$ 25	N $=$ 25	N $=$ 24	N $=$ 24
Mean accuracy (%)	66	54	50.69	48.55
	(SD $=$ 21.77)	(SD $=$ 20.85)	(SD $=$ 11.5)	(SD $=$ 16.6)
Significance	${p} = {.001}$	${p} = .347$	${p} = {.770}$	${p} = {.680}$
	95 % CI [7, 25]	95 % CI [$-$4.6, 12.6]	95 % CI [$-$4.2, 5.6]	95 % CI [-8.6, 5.4]
Effect size	${d} = {.750}$	${d} = {.194}$	${d} < {.001}$	${d} = -{.087}$
Explicit learners	4	3	0	7
100 ms				
No.	N $=$ 24	N $=$ 25	N $=$ 25	N $=$ 23
Mean accuracy (%)	53.82	56	49	57.99
	(SD $=$ 17.54)	(SD $=$ 20.48)	(SD $=$ 8.09)	(SD $=$ 15.63)
Significance	${p} = {.297}$	${p} = {.156}$	${p} = {.543}$	${p} = {.017}$
	95 % CI [$-$3.6, 11.2]	95 % CI [$-$2.5, 14.5]	95 % CI [$-$4.3, 2.3]	95 % CI [$-$4.5, $-$4.4]
Effect size	${d} = {.218}$	${d} = {.371}$	${d} = {.060}$	${d} = {.516}$
Explicit learners	9	4	0	3
Total mean accuracy (%)	60.03	55	49.83	53.37
	(SD $=$ 20.55)	(SD $=$ 20.48)	(SD $=$ 9.84)	(SD $=$ 16.63)

To compare performance across the 6 Conditions we ran a Generalized Linear Mixed Model analysis (using IBM SPSS version 20.0.0), with Accuracy (correct responses, meaning correct rejections of ungrammatical, and correct acceptance of grammatical test strings) as a (binomial) dependent variable. We introduced Subjects as a random factor, and Acoustic Condition (Emphasized, Lexical, Functional), Pause version (100, 250 ms), and the interaction Acoustic Condition $\times$ Pause version as fixed factors. We also introduced Language (L1, L2) as a fixed factor, to control for stimulus-specific biases (the possibility that certain *a_b* combinations were inherently easier to learn than others), and the interaction Acoustic Condition $\times$ Language (because we used the same *a* / *b* words but with different perceptual properties in each Condition, we wanted to control for the possibility that the manner of recording of these different stimuli had introduced a bias for certain *a_b* combinations in some but not all of the Acoustic Conditions). There was a significant effect of Acoustic Condition ($F (2, 1.767) = 4.161, p = .016$), with Bonferroni planned comparisons yielding a near-significant difference between the Emphasized and Functional Conditions ($t (1) = 1.884, p = .06, 95\,\%\,\, \hbox {CI}\,\, [-20, 99]$) but no other main effects or interactions (no effect of Language, $p = .914$, or Pause, $p = .185$, and no Acoustic $\times$ Pause, $p = .115$ or Acoustic $\times$ Language, $p = .213$ interaction). The difference in performance between the Emphasized and Functional Condition, therefore, approached significance; the Lexical Condition showed accuracy rates in between those of the Emphasized and Functional Conditions, respectively, and not significantly different from either. This pattern of results suggests that NAD-learning decreased with the decrease in acoustic prominence of the dependent elements.

### Discussion

Experiment 1 tested learning of NADs in three different Acoustic Conditions and two different Pause versions; intervening *X* elements were kept the same throughout Conditions, while the perceptual properties of dependent elements *a* and *b* in *aXb* strings were varied (ranging from Emphasized, Lexical-sounding and Functional-sounding). We obtained a significant effect of Acoustic Condition, reflecting a marginally significant improvement in performance in the Emphasized Condition with respect to the Functional Condition. This pattern of results is consistent with the claim that adult participants were influenced by the acoustic *prominence* of the dependent tokens, such that the more perceptually prominent the elements, the easier the dependencies were to learn. Only the Emphasized Condition with 250 ms pauses, which resembled the stimuli of Gómez (2002), yielded learning that was significantly above chance. Note that the learning effect in this condition was lower than the general learning performance in Gómez (2002). As mentioned above, in the current study we changed important aspects of the methodology, by promoting incidental over explicit learning, and testing participants’ ability to generalize these dependencies to novel strings (with novel intervening *X*s). These changes were implemented to more realistically reflect the natural language learning situations we are trying to model, but they may also have rendered the task more difficult.

The fact that we obtained significant learning in the Emphasized Condition only in the 250 ms Pause Version, and not in the 100 ms Version may suggest that participants found it easier to exploit the perceptual cues in the condition with the more naturalistic prosody; however, the analysis of the data did not yield a significant Acoustic Condition * Pause Version interaction, therefore we have no basis to draw a conclusion about the role of prosody in NAD-learning.

Verbal reports completed after the experiment revealed that some participants in the Functional Conditions did not segment the *aXb* strings as intended. Nine participants in the 250 ms version and four in the 100 ms one reported that they had perceived familiarization strings as having the syllable structure 1–1–2 (as opposed to the correct 1–2–1), suggesting the possibility that they had segmented the string-final element *b* as the initial element of the subsequent string (*baX*, as opposed to *aXb*). Participants in the Lexical and Emphasized Conditions never reported this segmentation: the participants in those conditions who recalled the structure of the strings unanimously reported the correct 1–2–1 structure.

This mis-segmentation introduced a confound in two Functional Conditions that renders the results unreliable. We attributed mis-segmentation to the prosodic properties of the *b* elements: because these elements were recorded as phrase-initial nonce determiners, their prosodic contour was not appropriate for a string-final position. Thus, the prosodic contour of our *aXb* strings was unnatural, or rather the pause segmentation cues conflicted with the prosodic segmentation cues. As a consequence, some participants ignored the long, 750 ms pauses between strings (that separated a *b* token from a subsequent *a* token), and combined the last word from a string to the first two words from the next string in a single prosodic phrase (*ba*X).^3^ $$^,$$ 4 We wanted to eliminate the possibility that participants in the Functional Conditions performed poorly solely because of the unnatural prosodic contour of the familiarization stimuli, and its interference with the correct segmentation of the strings. For this purpose, we ran a new version of the Functional Conditions, in which the prosodic contour of the *aXb* strings facilitated their segmentation and did not conflict with the pauses that delimited them.

## Experiment 2

### Participants

We recruited 51 participants (5 male, age range 18–42, mean age 22) in the same way as before; 4 participants were excluded, 3 due to technical problems and one for familiarity with research on NAD-learning. Of the remaining 47, 24 were assigned to the New Functional Condition with 100 ms pauses, and 23 to the New Functional Condition with 250 ms pauses.

### Materials

Tokens of the artificial grammar were re-recorded in similar fashion as before: the *X* items were recorded in the same carrier-sentence (4). For the *a* and *b* tokens, we chose the morpho-syntactic dependency between the neuter determiner *het* and the diminutive suffix *–(t)je* in Dutch as a model, and recorded the *a* and *b* tokens as the determiner and suffix respectively:

**Table:** 

(6) Ik zie **het** zebra’**tje**.
I see the zebra.DIM
e.g. Ik zie **tep** zebra’tje
Ik zie het zebra**lut**.

**Table 4:** Acoustic measures of the a/b tokens for Experiment 2

		Functional–new recordings		
		Mean pitch (Hz)	Mean amplitude (dB)	Duration (s)
a	TEP	219.9	80.4	0.176
	SOT	220.8	77.3	0.277
	RAK	209.8	76	0.25
b	LUT	188.9	78.7	0.305
	JIK	184.4	79.2	0.27
	TOEF	191.6	74.3	0.346
	Mean	202.6	77.7	0.2707

Note that while other measurements are comparable to the old recordings, the duration of the new tokens is longer, particularly of the *b* tokens—this lengthening is a natural consequence of their phrase-final position

We analyzed the *a* / *b* tokens acoustically, the same way as before (see Table 4). The testing procedure was identical to Experiment 1.

### Results

We compared the results in each Pause version of the New Functional Condition with chance (50 %) performance: a One-Sample *t* test on the mean Accuracy scores (percentage correct responses per participant) for the New Functional Condition with 250 ms pauses revealed no significant learning effect ($p = .680, 95\,\%$ CI $[-8. 6, 5.4]$, Cohen’s $d = -.087$), with participants clearly scoring at chance (M $=$ 48.55 %, SD $=$ 16.6). The mean Accuracy score in the New Functional Condition with 100 ms pauses was 57.99 % (SD $=$ 15.63). As a one sample Kolmogorov–Smirnov Test showed that the accuracy scores were not normally distributed ($p = .043$), we ran a non-parametric test (One-Sample Wilcoxon Signed Rank Test) on the mean Accuracy scores, which showed that performance was significantly better than chance (Median $=$ 0.58, SE $=$ 18.78, Z $=$ 2.396, $p = .017$) with a Cohen’s *d* of .516.

We compared the new Functional Conditions with the old Emphasized and Lexical Conditions in a Generalized Linear Mixed Model as before (Acoustic Condition, Pause, Language, Acoustic $\times$ Pause and Acoustic $\times$ Language as fixed factors, and Subject as random factor). There was no significant effect of Pause ($p = .921$), Language ($p = .487$), Acoustic Condition ($p = .175$), or Acoustic Condition $\times$ Language interaction ($p = .236$), but there was a significant Pause x Acoustic Condition interaction ($F (2, 1.743) = 3.819, p = .022$). Whereas participants in the Emphasized Condition performed better in the 250 ms Pause Version than in the 100 ms Pause Version, the Functional Condition showed better performance in the 100 ms Pause Version than in the 250 ms one, suggesting that the prosodic properties of the strings modulated the effect of acoustic cues on NAD-learning.

None of the participants reported an incorrect segmentation strategy. Together with the improvement in learning in the New Functional Condition with 100 ms pauses this suggests that mis-segmentation was no longer an impediment to learning in Experiment 2.

We also pooled the data from Experimens 1 and 2 and ran a two-tailed Pearson correlation test to verify whether participants’ confidence in their responses correlated with their actual performance. We obtained a low but significant correlation between the confidence ratings on a 1–7 scale and the accuracy rates per participant $(\hbox {r} = .198, p = .006)$. Some participants reported being aware of the presence of a dependency between the first and last word of the strings: Table 3 shows, for each condition, how many participants reported awareness of a dependency (some, but not all of these obtained a 100 % accuracy score). When we excluded the participants who reported awareness of a pattern, the correlation between performance and confidence ratings was non-significant, both in the overall dataset $(\hbox {r} = -.068, p=.385)$ and in the individual conditions, suggesting that the overall effect was carried exclusively by the high confidence ratings of those participant who also reported awareness of the NAD. None of the participants that became explicitly aware of the pattern reported intentionally looking for patterns in the input: instead, all of them reported that at some point in the familiarization phase they suddenly became aware of dependencies, due to the frequent occurrence of the short (dependent) nonce-words. In general, awareness (of the properties of the familiarization language) ranged from failing to indicate the correct number of words in a string (3), to indicating the syllabic structure of an *aXb* string (1–2–1), to indicating some/all of the *a* / *b* words and their position in a string, and finally to identifying the presence of a dependency between the first and last word in an *aXb* string and being able to recall none, some or all of the dependencies. All participants colored a substantial part of the mandala, suggesting that all participants were engaged in the coloring task while listening to the language at familiarization.

### Discussion

We retested participants’ learning of NADs in the Functional Condition with familiarization strings exhibiting a rising-falling pitch contour: we recorded the stimuli in the same way as before, with the minor difference that the *b* elements were recorded as phrase-final functors in a natural morpho-syntactic template. This allowed the *aXb* strings in the familiarization to have a rising-falling pitch contour similar to prosodic phrases in natural languages, and eliminated the risk of mis-segmentation.

Performance in the Functional Conditions improved selectively: participants’ accuracy in judging the test items was significantly above chance when intra-stimulus pauses were 100 ms, but were at chance when the pauses were 250 ms. Thus, the results for the retested Functional Condition contradict our initial interpretation of Experiment 1: learners are able to detect dependencies even when the dependent elements are less salient, provided they are prosodically distinct from the intervening material. These results are in line with the hypothesis put forth by Newport and Aslin (2004), that NADs are detected through a mechanism of Gestalt perception. By contrast with the retested Functional Condition, participants in the Emphasized Conditions performed above chance with 250 ms pauses, but not with 100 ms pauses. This result is not unexpected. We assumed that if the within-string pauses played a role, the 100 ms pauses would facilitate learning in the Functional Condition, because the functional-sounding *a* / *b* tokens had the prosodic status of clitics (Selkirk 1996), and would therefore sound more natural if isolated by shorter pauses;^5^ we also assumed that 250 ms within-string pauses would be more appropriate for the stimuli in the Emphasized Condition, as in the latter the *a* / *b* tokens had the status of highly emphasized words, which in natural speech can often be separated by a perceivable pause from the rest of the sentence.

The above-chance performance in the Emphasized 250 ms and the New Functional 100 ms Conditions suggests that NAD-learning is optimal (consistent with Hypothesis 2) when the dependent elements are perceptually distinctive but integrated into a prosodically natural contour. The significant Pause by Acoustic Condition interaction suggests that prosody is crucial to NAD-learning: learners seem to employ the acoustic cues in the input to establish the prosodic status of each element in a string, and then apply Gestalt principles *at the prosodic level of organization* to aid the computation of co-occurrence statistics between prosodically (rather than acoustically) similar elements. If prosodic cues are conflicting (pauses separating words are not consistent with the prosodic status of the word), the computation is rendered more difficult.

Finally it is important to note that in Experiments 1 and 2 participants reached varying levels of awareness of the pattern in the input. One suggested measure to assess whether there is implicit learning (Dienes 2007) is to check for correlations between participants’ assessment of their performance, and their actual performance: if this correlation exists, then participants have at least some explicit awareness that their answers are being guided by knowledge of the structure of the language. When we excluded participants who reported awareness of the rules of the language, we found no correlations between participants’ accuracy rate and their overall confidence in their performance, suggesting that if they developed any sensitivity to the rules of the language, they were unlikely to be aware of it.

A question that arises is whether the fact that some participants became aware of the dependencies threatens the validity of the findings. We would argue that this is not the case. Firstly, all participants received the same instructions, and all explicit learners reported that they had obeyed the instructions, listening to the language passively and only noticing the dependencies (after hearing a substantial amount of input) due to their recurrence in different strings. All participants were exposed to the language in incidental learning conditions, unaware that they were expected to acquire the structure of the language, or what type of structure it was; all were given a simultaneous task and none were warned about the existence of a subsequent test phase, so as to discourage memorizing the input or looking for patterns. Therefore, there is nothing to indicate that the procedure differed across participants. Why then would the outcome, namely the amount of implicit/explicit knowledge *derived* from the same incidental learning process differ per participant? The literature (see Reber 1989 and references cited therein) emphasizes that implicit learning is more likely to occur with complex grammar, made up of multiple rules. In our study, we tested the acquisition of a simple rule: the dependency between the first and last words in a string,^6^ and thus allowed for the possibility that participants would derive explicit knowledge of that rule if their sensitivity to it exceeded a certain threshold. Note that the distribution of explicit learners was not even across conditions, but there were more explicit learners in the conditions predicted to induce above-chance performance (9 in the Emphasized Condition with 250 ms pauses and 7 in the New Functional Condition with 100 ms pauses) than in the rest of the conditions (3–4). Instead of assuming that participants in these specific conditions happened to be less likely to follow the instructions, we propose that it is more likely that these conditions promoted explicit awareness of the rules by facilitating the detection of those rules.

## General Discussion

This study investigated the role of perceptual factors in the process of learning dependencies between non-adjacent elements in spoken input. We asked *whether* perceptual factors can influence a distributional learning mechanism like NAD-learning at all, and if so, *how* perceptual cues might affect NAD-learning. Participants’ ability to learn dependencies between non-adjacent elements in an artificial language (*aXb*) was tested by eliciting their endorsements for novel strings (with a novel intervening $X'$) with either grammatical $(a_{i} X'b_{i})$ or ungrammatical $(a_{i} X'b_{j})$ dependencies. Participants’ learning performance, quantified as their accuracy in accepting grammatical strings and rejecting ungrammatical ones, was modulated by the differences in prosodic properties of the dependent elements. The results of Experiment 1 suggested that learning declined when perceptual cues rendered dependent elements less prominent: participants acquired dependencies between highly acoustically salient words (Emphasized Condition), but were not sensitive to these dependencies when the target words were not particularly salient (Lexical Condition), or when the target words were phonetically ‘reduced’ (Functional Condition). However, performance in the Functional Condition may have been affected by a confounding factor, namely the unnatural prosodic contour of our stimuli leading to an erroneous segmentation strategy. When we eliminated this confound, the picture we obtained was quite different. Participants were successful in detecting dependencies both between highly prominent (Emphasized), and between perceptually ‘reduced’ elements (Functional), but only when the strings had specific prosodic properties: we obtained reliable discrimination of grammatical and ungrammatical strings in the Emphasized Condition only in the 250 ms Pause version, and in the Functional Condition only in the 100 ms version.

It is important to note that our conclusions rely on a comparison between the findings in Experiments 1 and 2. Stimuli for Experiment 2 were recorded afresh, under the same conditions and with the same speaker instructions so that they matched the stimuli in Experiment 1 as closely as possible apart from the necessary manipulations. Furthermore, the nature of the task in our experiments demands a between-subjects design. Thus, Experiments 1 and 2 differed in stimuli, participants, and time of data collection, despite our best efforts to minimize those differences (by ensuring the stimuli were comparable in acoustic properties, by recruiting and assigning participants to conditions in the same way, etc.). While the Generalized Linear Mixed Model we used included individual variation as a factor, it did not take into account the different set of stimuli used in Experiment 2, and thus the results it yields should be interpreted with caution.

### NAD-Learning Guided by Gestalt Principles of Perception

If NADs are acquired based on a mechanism that simply computes co-occurrence probabilities between non-adjacent units, why would this mechanism be affected by the acoustic/prosodic properties of these units? Our results pattern with the findings of Newport and Aslin (2004), Creel et al. (2004) and Gebhart et al. (2009), who propose that NAD-learning is facilitated in contexts where Gestalt principles of perception allow the dependent elements to be somehow grouped together, on a separate representational level, based on their perceptual distinctiveness from the intervening material. Thus, participants in the Emphasized Condition and the Functional Condition could have detected the dependencies due to an initial bias to group them together—this bias was due both to the difference in phonetic/prosodic properties between the target elements and the intervening material, and to the perceptual similarity between the target *a* and *b* elements. Hence, the dependent elements in the Lexical Condition were not distinctive enough to be grouped together, as *a*, *b* and *X* elements all had the same prosodic status.

In addition to our finding that dependency-detection may be facilitated by Gestalt principles of perception, we also found evidence that the domain where these Gestalt principles could apply is the linguistic prosodic domain. Two important aspects of our findings support the conclusion that it is at the level of prosody that elements are grouped together based on similarity. Firstly, in Experiment 2, a significant interaction was found between Pause Version and Acoustic Condition, suggesting that it was not only the acoustic properties of the dependent elements that affected learning, but also the way in which these elements were integrated into the prosodic contour of the strings. Participants performed better where the inter-word pauses matched the prosodic status of the dependent elements (short, 100 ms pauses for functional-like nonce words—resembling prosodic clitics—and longer, 250 ms pauses for prosodically marked—emphasized—nonce words), suggesting that properly marking the prosodic status of elements was crucial to the learning mechanism.

Secondly, participants’ success in acquiring dependencies in the New Functional Condition with 100 ms pauses is interesting in itself. In Experiment 2 the *a* / *b* elements were recorded to fit into a natural phrasal prosodic contour. However, because they were no longer recorded in precisely the same slot in their carrier sentences, their acoustic properties now differed. While *a* elements had a rising pitch contour, *b* elements had a falling one; furthermore, because *b* elements were now phrase-final, they were subject to final lengthening, meaning the duration of *b* elements was longer than that of *a* elements, as can be observed in Table 4. If *a_b* dependencies in this experiment were detected based on the acoustic similarity of *a* / *b*, then the difference in duration and pitch contour between $a/\hbox {b}$ in the New Functional 100 ms Condition should not have facilitated the detection of the dependencies. Instead, learners seem to have abstracted away from the acoustic differences between the *a* and *b* classes and categorized them both as prosodic clitics, with different positions in a larger (phrasal) prosodic unit.

Our findings are also in line with the claim in Newport and Aslin (2004) that NAD-learning is constrained in ways compatible with the properties of natural languages. In our experiments, one of the two Conditions where participants showed above-chance performance was the one that emulated the prosodic properties of morpho-syntactic dependencies in many natural languages: the dependent elements were perceptually similar to functors, and were separated by minimal (100 ms) pauses from the intervening lexical-sounding nonce words, in strings that aimed to resemble the rising-falling pitch contour of a natural phrasal unit. The fact that this condition enabled learning, when the Lexical Condition did not, suggests that the prosodic and perceptual cues that natural languages exhibit are well-suited for the detection of co-occurrence patterns between functional morphemes.

Note however, that while both the Emphasized 250 ms and the New Functional 100 ms Conditions produced above-chance accuracy scores, the effect sizes reported in Table 3 show a somewhat smaller effect in the New Functional 100 ms Condition: it may be, therefore, that although prominence of the dependent elements is not a crucial factor to NAD-learning, it does facilitate the detection of dependencies. The potential importance of acoustic prominence as a perceptual cue to NADs may indicate that the properties of function words in natural languages do not, perhaps, make them *ideal* for learning dependencies between them. However, it does not detract from the core finding that non-adjacent dependencies are learned even when dependent elements are perceptually less salient than the intervening material, and that dependencies emulating the properties of natural-language morpho-syntactic dependencies are learned reliably in an artificial grammar learning setting.

### Questions for Future Research

An important point to note is that Newport and Aslin ’s (2004) proposal states that dependencies are learned not only based on the perceptual distinctiveness of the dependent elements but also, crucially, on their mutual perceptual *similarity* which allows them to be computed on the *same* separate perceptual tier. In our study the perceptual similarity between *a* and *b* in *a_b* dependencies was a given in all conditions, and was therefore not independently investigated. The question whether perceptual similarity between dependent elements is crucial to the detection of dependencies needs to be investigated in a separate study, bearing in mind that perceptual/prosodic difference between dependent elements may also arise in natural languages. For instance, in a language like French which assigns prominence to a phrase-final syllable, a dependency like subject-verb agreement in *Nous* *chant* *ons* (We sing.$\underline{1^{\mathrm{st}}\hbox {pl}}$) is instantiated between a non-prominent pronoun and a metrically prominent agreement suffix. It is important to look into the potential effects that such a configuration could have on the ability to learn the dependency between them.

It is equally important to point out that participants in these experiments were adults, who might have treated the input as a natural language, and may have drawn from their already established experience of natural language(s) the notion that perceptually distinct/reduced elements are generally likely to entertain (morpho-syntactic) dependencies. The stimuli in these experiments were Dutch-sounding nonce words, recorded by a Dutch native speaker; the New Functional Condition with 100 ms pauses was meant to best emulate morpho-syntactic dependencies in Dutch, which was the native language of the participants. It is, therefore, not far-fetched to assume that participants might have been detecting dependencies in the artificial language they heard more easily based on their (perceptual) similarity to dependencies in their own native language.

If adults’ detection of the dependencies in the Functional Condition was facilitated by their experience of natural languages exhibiting similar kinds of dependencies, then this may represent a confound in our study, and obscure the way the NAD-learning mechanism functions in the absence of other biases. One way to eliminate this bias would be to test learning of dependencies between perceptually reduced elements in a non-linguistic domain, either visual or auditory. Creel et al. (2004), as well as Gebhart et al. (2009) showed that NAD-learning can be extended to patterns between non-linguistic elements, and that NAD-learning in the non-linguistic domain may also be driven by Gestalt principles of perception. Participants’ sensitivity to non-adjacent patterns between perceptually reduced elements in a string of tones or noises would prompt the conclusion that the results we find in this study may not be entirely determined by linguistic experience, but may reflect a more general property of the NAD-learning mechanism.

Furthermore, the NAD-learning mechanism may be laid bare better in a study with participants who are not (or less) biased by knowledge of / experience with natural language dependencies: infants. In order to establish whether participants in our experiment were relying on their knowledge of natural languages, or purely on prosodic cues, this study should be replicated with infants, who are capable of dependency-learning but do not yet show knowledge of dependencies in their own language. Note that although behavioral evidence suggests the NAD-learning mechanism emerges around 15 months (Gómez and Maye 2005), neurophysiological evidence suggests that sensitivity to non-adjacent patterns arises as early as 4 months (Friederici et al. 2011). Friederici and colleagues exposed German 4-month-old infants to Italian sentences containing two morpho-syntactic dependencies (*La sorella* *sta* *cant* *ando*, ‘The sister is singing’ vs. *Il fratello* *puo* *cant* *are*, ‘The brother can sing’) and measured ERP response to sentences containing grammatical vs. ungrammatical dependencies (*La sorella* *sta* *cant* *ando* vs. *La sorella *sta* *cant* *are*). Infants showed a significant positivity 640–1040 ms after the onset of the suffix, when the suffix was mismatched with the auxiliary compared to when the two were well-matched. This positivity increased across learning blocks, suggesting that infants were gradually developing sensitivity to the morpho-syntactic dependency, in a language they had never heard before.

It is unclear, however, at what age the child should/can be considered to have no relevant knowledge of NADs in the native language. Indirect evidence can be obtained by comparing NAD-learning at different ages: if younger infants around the age of 15 months learn NADs only in the Emphasized, and not the Functional Condition, whereas older infants (e.g. 18–24 months) can learn dependencies in both Conditions, it could be argued that the ability to detect NADs when the dependent elements are perceptually non-salient is not an intrinsic property of NAD-learning, but is an acquired ability. Infant research is required to disentangle potential factors contributing to the adult results reported.

### Conclusion

We investigated the role of perceptual cues to NAD-learning by studying the effect of prosodic manipulations on the acquisition of remote dependencies following exposure to an artificial grammar. We showed that learners can detect non-adjacent dependencies and generalize them to novel strings only under particular conditions. We extended previous findings by presenting evidence that detection of dependencies between non-adjacent elements is driven by Gestalt principles of perception, grouping together similar elements on a separate level of analysis; we further argued that this is likely to be a level of prosodic analysis, as learning was facilitated when cues to the prosodic status of the individual elements (either acoustic properties of the elements or the length of pauses separating them from adjacent words) were non-conflicting. Our findings suggest that in natural languages, where prosodically unmarked/clitic-like functional morphemes alternate with more prosodically marked lexical morphemes, patterns between functional words can be easily captured due to Gestalt principles of perception.

## Footnotes

1 By contrast, Endress and Bonatti (2006) propose that a rapid mechanism computing dependencies between structural positions precedes the slower, statistical computation of correspondences between individual tokens.

2 Note that stimuli for artificial grammar learning experiments like Gómez (2002) are cross-spliced: any token (*a*, *b*, or *X*) is ‘cut’ from the original recording and ‘pasted’ into different strings where it can be adjacent to different words. This makes it difficult to introduce coarticulation at the point of transition between two different words in a string. As our primary interest was not coarticulation cues, we decided to introduce minimal within-string pauses to circumvent the need for coarticulation altogether.

3 Another way participants might have obtained the 1–1–2 syllable structure is by simply grouping the final syllable of the *X* elements together with the *b* syllable; we reject this possibility for two reasons. First of all, the second syllable of the $\times$ elements was metrically weak, and it is unlikely that a weak syllable would be segmented from a strong syllable (the first syllable in *X*), only to be combined with another weak syllable (*b*) into a bisyllabic word. Secondly, participants in this condition alone did not show a bias towards accepting test items, as they did not conform to the structure 1–1–2; if they had simply mis-segmented the *X* they would have made the same segmentation error at test—they would not have reported a different, 1–2–1 structure for test items, nor would they have been led to reject test items more than in other conditions.

4 Note that the same problem does not apply to the Emphasized condition, where the *a* and *b* elements were spliced from recordings where they were strings-medial, and, respectively, string-final. Thus, while the *a* tokens had a rising intonation, the *b* tokens had a (rising)-falling pitch contour, which conferred an overall natural rising-falling intonation contour to the aXb strings. In the Lexical Condition too, lexical *a* / *b* tokens had a rising-falling pitch contour which conferred the aXb strings a rising-falling contour.

5 Note, furthermore, that a 100 ms pause, together with the high combinatorial properties of the *a* and *b* tokens, was sufficient to facilitate segmentation of the individual nonce words in the *aXb* strings: in their verbal reports participants often recalled individual words, particularly *a* and *b* tokens. Furthermore, Peña et al. (2002) evidenced an effect of segmentation of *aXb* strings by introducing smaller, imperceivable, pauses of only 20 ms. Finally, the fact that participants identified grammatical dependencies in novel $aX'b$ strings suggests that they were parsing *a* and *b* tokens as separate units from the *X*s in the familiarization.

6 Note that merely because this rule is *simple*, it is not also necessarily *easy* to acquire, as our results indicate. Simplicity in Reber’s terms is the property of systems that could be very easily learned with explicit instruction
